# Research hotspots and trends in biliary tract cancers immunotherapy are revealed by a bibliometric study spanning the years 2014 to 2024

**DOI:** 10.1097/MD.0000000000046834

**Published:** 2026-09-25

**Authors:** Yanyan Miu, Cuiling Zhang, Yan Niu, Peirong Wang, Zhongbiao Nie

**Affiliations:** a Department of Oncology, Shanxi Bethune Hospital, Shanxi Academy of Medical Sciences, Third Hospital of Shanxi Medical University, Tongji Shanxi Hospital, Taiyuan, China; b Department of Pharmaceutical, Shanxi Bethune Hospital, Shanxi Academy of Medical Sciences, Third Hospital of Shanxi Medical University, Tongji Shanxi Hospital, Taiyuan, China.

**Keywords:** bibliometric analysis, biliary tract cancers, gemcitabine, growth factor receptor, immunotherapy, research trends, tumor microenvironment, tumor-infiltrating lymphocytes

## Abstract

**Background:**

This bibliometric study aimed to map the global research landscape of immunotherapy for biliary tract cancers (BTC) from 2014 to 2023, identifying key contributors and emerging trends.

**Methods:**

We analyzed 481 English-language articles retrieved from the Web of Science Core Collection (2014–2023) using CiteSpace. Parameters included coauthorship, co-citation, keyword co-occurrence, and burst detection.

**Results:**

Annual publications surged by 80% in 2021 versus 2020. China dominated output (50% of publications), with Fudan University as the top institution. Fan Jia was the most prolific author (7 papers). High-frequency keywords included gemcitabine (first-line chemotherapy), tumor microenvironment, and T cells. Burst keywords indicated emerging foci: tumor-infiltrating lymphocytes and growth factor receptor (e.g., HER2/ERBB2). The most co-cited study (Piha-Paul et al, 2020; 106 citations) reported pembrolizumab efficacy in BTC.

**Conclusion:**

Research accelerated post-2020, driven by clinical trials. China, Fudan University, and Fan Jia are central to BTC immunotherapy research. Future priorities include targeting HER2 and the tumor microenvironment.

## 1. Introduction

A diverse and aggressive category of tumors that can arise from the gallbladder, the distal biliary tree, or the intrahepatic or extrahepatic epithelium.^[1]^ The prevalence varies by region; in the United States and Europe, it is regarded as uncommon, with 1 to 3 instances per 1,00,000 people, whereas in China, Japan, Korea, and Thailand, it is a serious health issue, with 5.7 to 85 cases per 1,00,000 people^[1-4]^. Radical surgery is currently the only therapy that has the potential to be curative. With a high risk of recurrence and an 8% to 30% 5-year survival rate, only 10% to 15% of tumors upon diagnosis may be surgically treated.^[5,6]^ Cholangiocarcinoma has long been regarded as an orphan illness with few available treatments. Additionally, immunotherapy has significantly altered the treatment arsenal for a number of cancers, turning a disease with a bad prognosis and metastases into 2 that can be cured in some situations.^[7,8]^

Understanding BTC immunotherapy research’s present condition and hotspots is difficult due to the field’s rapid evolution. There was no literature available to comprehensively analyses the published relevant material, despite the fact that an increase in published studies has led to major advancements in immunotherapy for patients with BTC. Bibliometrics (CiteSpace) is the quantitative analysis of a vast amount of literature in a certain subject of study using statistical and mathematical techniques. CiteSpace’s Web of Science Core Collection (WoSCC) makes quantitative scientific analysis possible.^[9-12]^.

## 2. Materials and methods

### 2.1. Source of literature

We entered the subject terms into the WoSCC kidney cancer AB= (“biliary tract cancers” OR “Bile Duct Neoplasms” OR “Common Bile Duct Neoplasms” OR “Gallbladder Neoplasms*” OR “cholangiocarcinoma*” OR “gallbladder carcinoma*”) AND AB=(“Immunotherapy” OR “Immunotherapies” OR “Immunotherapeutic” OR “immunology”). The database may only be searched in the English-language from January 1, 2014, until October 23, 2024. 1663 records were found in the literature search. Two authors (ZBN and YYM) independently examined the WoSCC database to locate relevant publications.

### 2.2. Analysis software

Version 5.6. R2 of the CiteSpace analysis program is available.^[13-15]^.

### 2.3. Download and import of data

After the results of the retrieved subject phrases were exported, the file format remained “plain text.”

### 2.4. Parameter setting

There are many steps in the process being examined: slicing time between 2014 and 2024; individually inspecting node types; application of a comprehensive set of 50 selection criteria; data trimming using the Pathfinder algorithm; and subsequent visualization which involves illustrating the merged network in a static cluster view.

### 2.5. Statistical methods

The yearly publications were analyzed, and data managed with Microsoft Office Excel 2021. After every work of literature was scientifically examined, important information was obtained, including co-cited references, authors, prominent nations, institutions, and keyword co-occurrences.^[12,16]^ Figure 1 depicts the comprehensive analysis flow.

**Figure 1.**
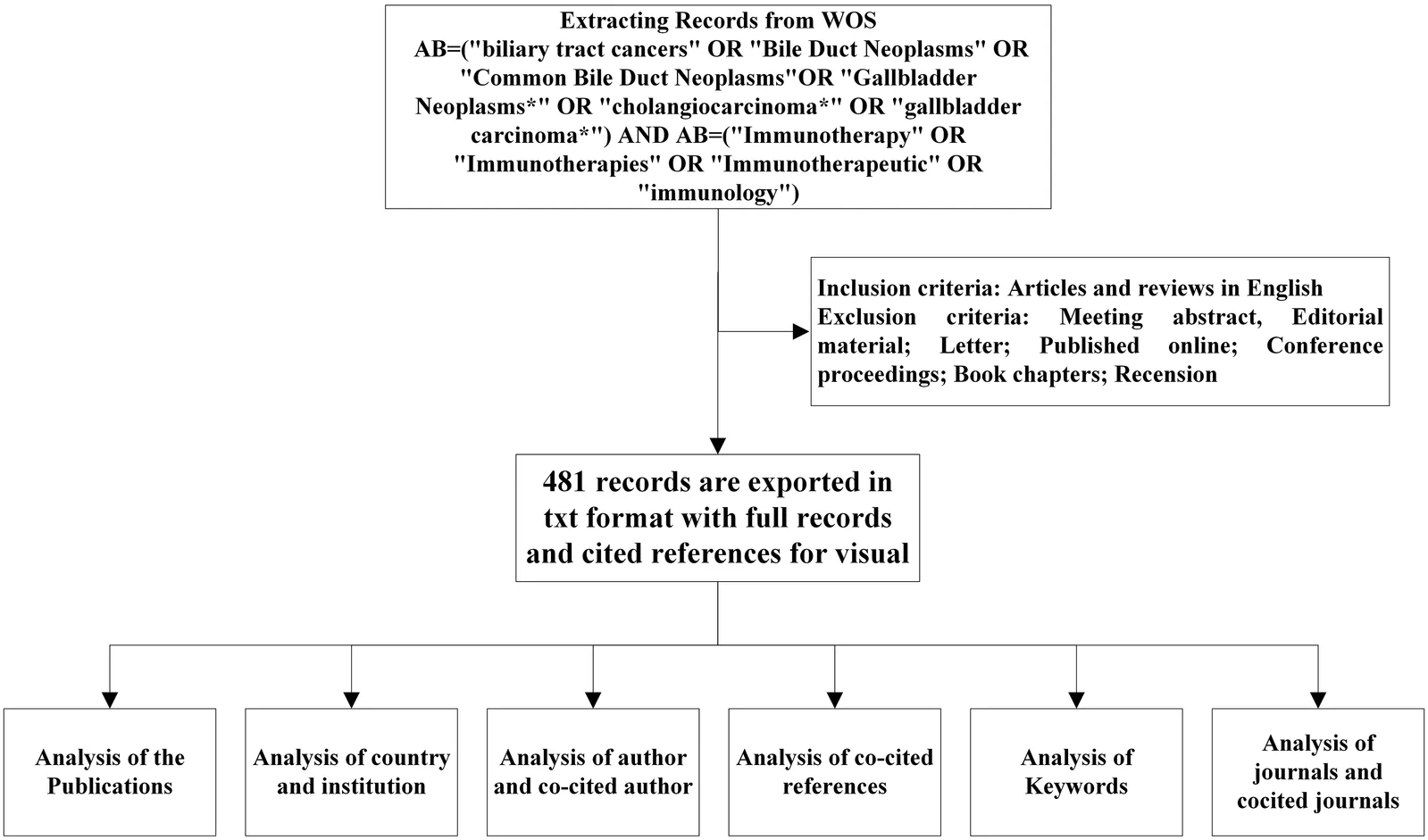
Analysis flow chart of immunotherapy for biliary tract cancers. WOS = Web of Science.

## 3. Results

### 3.1. Analysis of the publications

From 2014 to 2024, a total of 481 relevant articles were retrieved. The overall number of publications changed during the research period. Figure 2 shows the 2 stages of the inquiry: The first phase runs from 2014 to 2020, while the second phase runs from 2021 to 2024. In the second phase, development accelerated. 80 articles were published in 2021, the number of documents issued increased by 50% compared to 2020. These results imply that throughout the past 4 years, research on immunotherapy for BTC has become more significant.

**Figure 2.**
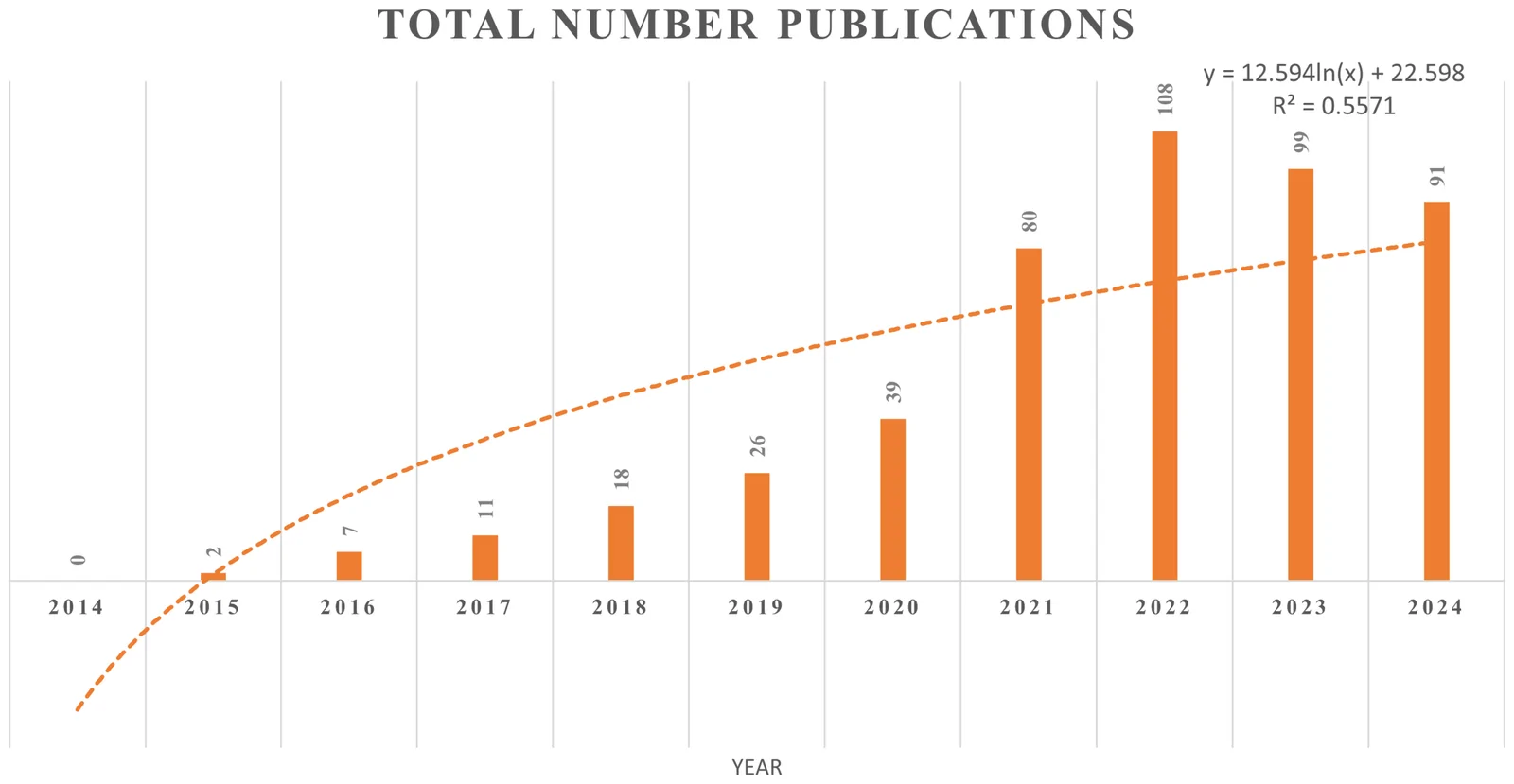
The number of immunotherapies for biliary tract cancers publications indexed by WOS from 2014 to 2024. WOS = Web of Science.

### 3.2. Analysis of countries and institutions

With an assembly of 615 relevant papers, a complete nation map representing the contributions from 43 different countries has been constructed and is shown in Figure 3. According to the research, the top 5 donors are the People’s Republic of China, United States, Italy, England, and France (Table 1). Thailand (0.59), Spain (0.58), Spain (0.48), Sweden (0.39), and France (0.34) were the nations that stood out in the ranking based on centrality (highlighted in purple). The number of publications from the People’s Republic of China accounted for half of all countries.

**Table 1:** Top 10 countries and institutions researching immunotherapy for biliary tract cancers.

Ranking	Country	Publications	Ranking	Institution	Publications
1	Peoples R China	212	1	Fudan University	28
2	Unites States	139	2	Harvard University	24
3	Italy	45	3	Mayo Clinic	22
4	England	29	4	Zhejiang University	20
5	France	22	5	Harvard Medical School	17
6	Germany	20	6	University System of Ohio	17
7	Thailand	16	7	Sun Yat Sen University	17
8	Spain	15	8	Shanghai Jiao Tong University	15
9	Japan	15	9	Huazhong University of Science & Technology	15
10	Netherlands	10	10	Institut National de la Sante et de la Recherche Medicale (Inserm)	13

**Figure 3.**
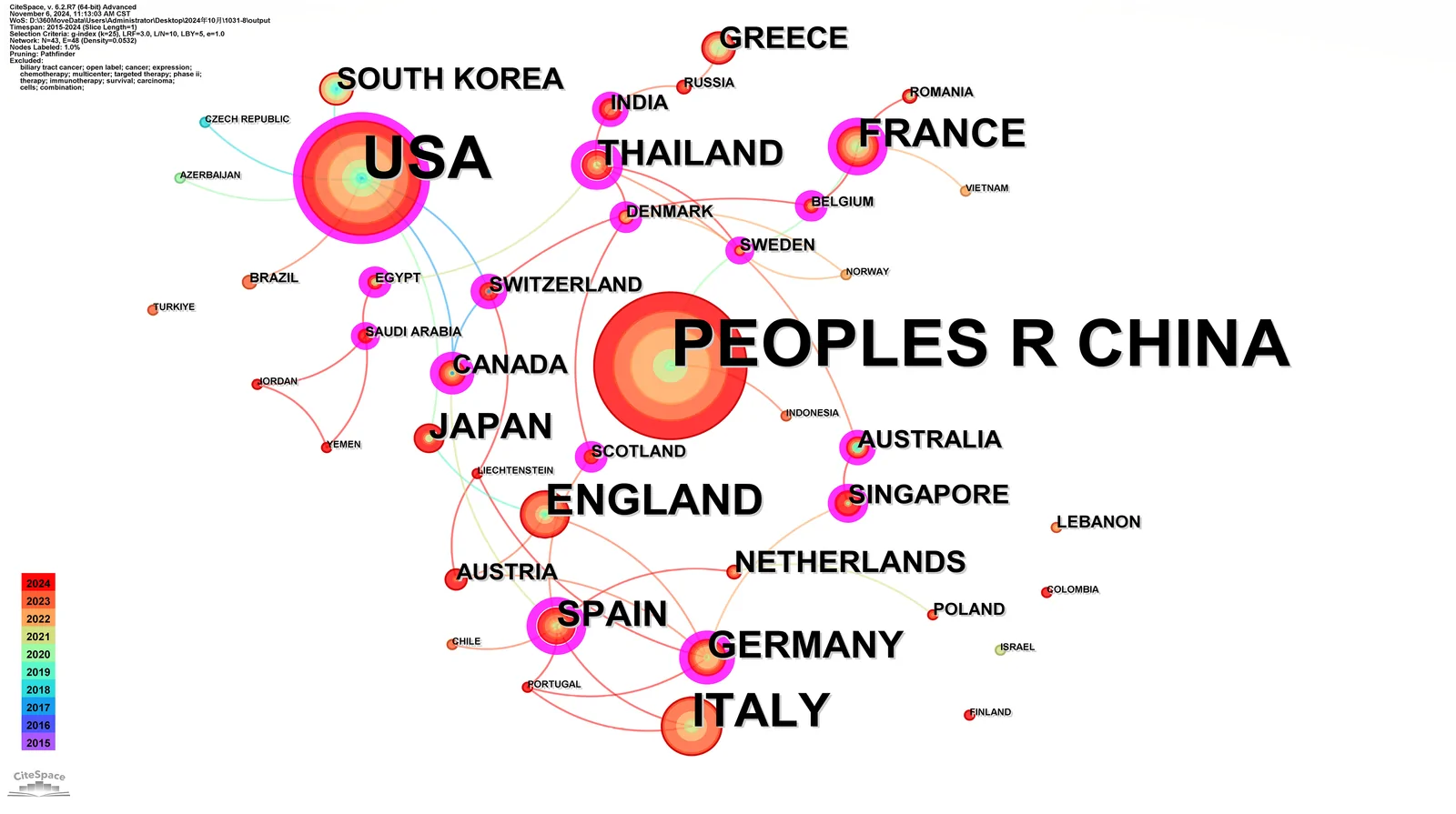
Analysis of the country map from 2014 to 2024.

The institution map, which has 219 connecting links and 366 nodes, is shown in Figure 4. Seven hundred fifty-eight papers from 219 different universities are included in the corpus of analysis. The top 5 contributing institutions are Fudan University, Harvard University, Mayo Clinic, Zhejiang University, and Harvard Medical School (Table 1). Weak connections between several institutions may also be observed, suggesting that they work closely together.

**Figure 4.**
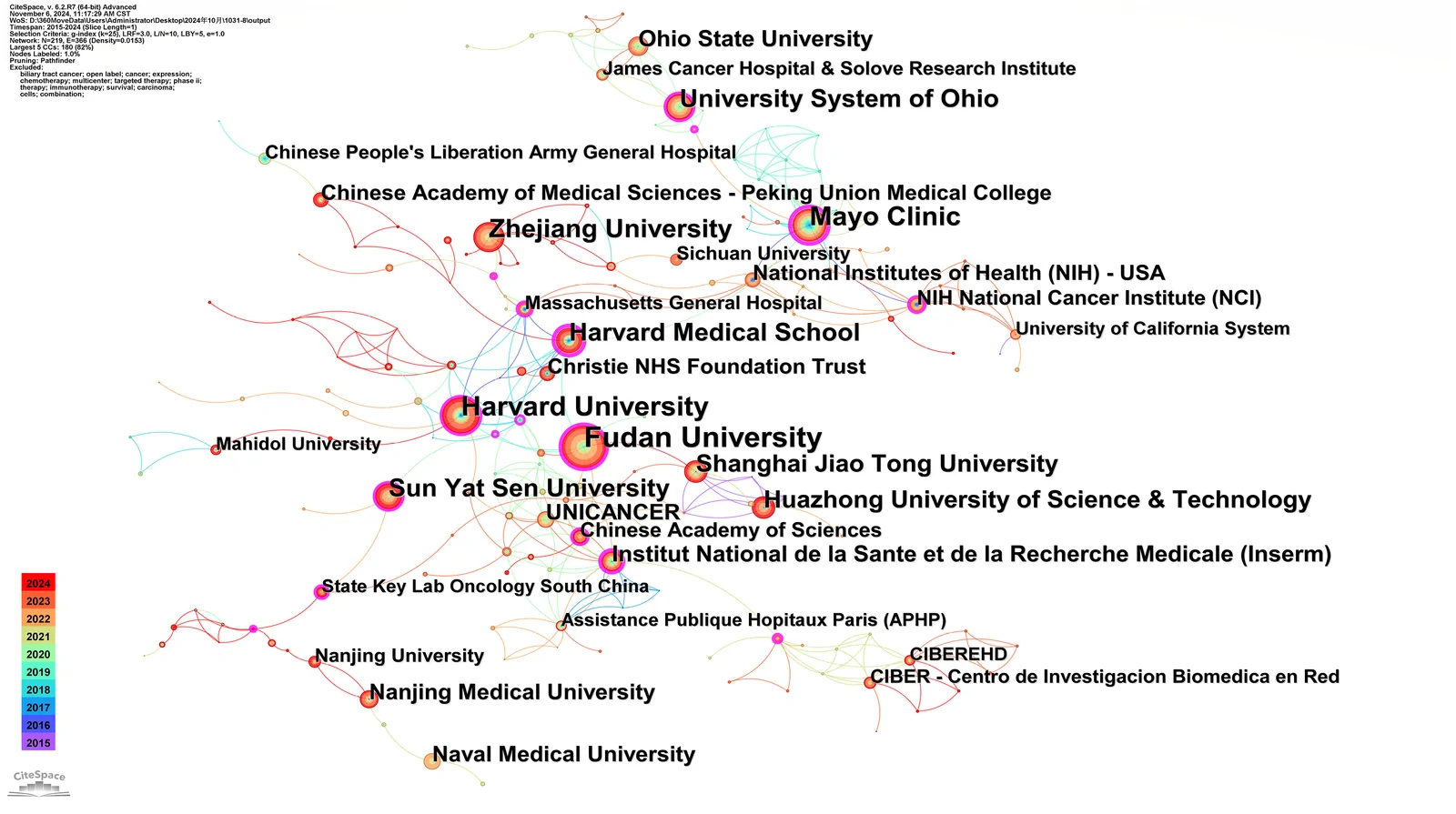
Institutional map researching immunotherapy for biliary tract cancers from 2014 to 2024.

### 3.3. Analysis of author

Seven hundred sixty-seven articles were published by 365 authors. The top 10 writers on this topic are subject-matter experts (Table 2). The generated map had 1219 nodes and 365 links (Fig. 5). Fan Jia, as the most published person (7), is employed at the Academician of Chinese Academy of Sciences, president of Zhongshan Hospital in China. Their group might offer clinical assistance to the field of BTC immunology. They are incredibly important in the business and have produced several publications with high impact factors.

**Table 2:** Top 10 authors in immunotherapy for biliary tract cancers in terms of centrality.

Ranking	Cited reference	Representative author (publication year)
1	7	Fan, Jia
2	7	Pawlik, Timothy M
3	6	Zhou, Jian
4	6	Lamarca, Angela
5	5	Goyal, Lipika
6	5	Gao, Qiang
7	5	Gores, Gregory J
8	5	Ilyas, Sumera I
9	5	Mahipal, Amit
10	4	Rizzo, Alessandro

**Figure 5.**
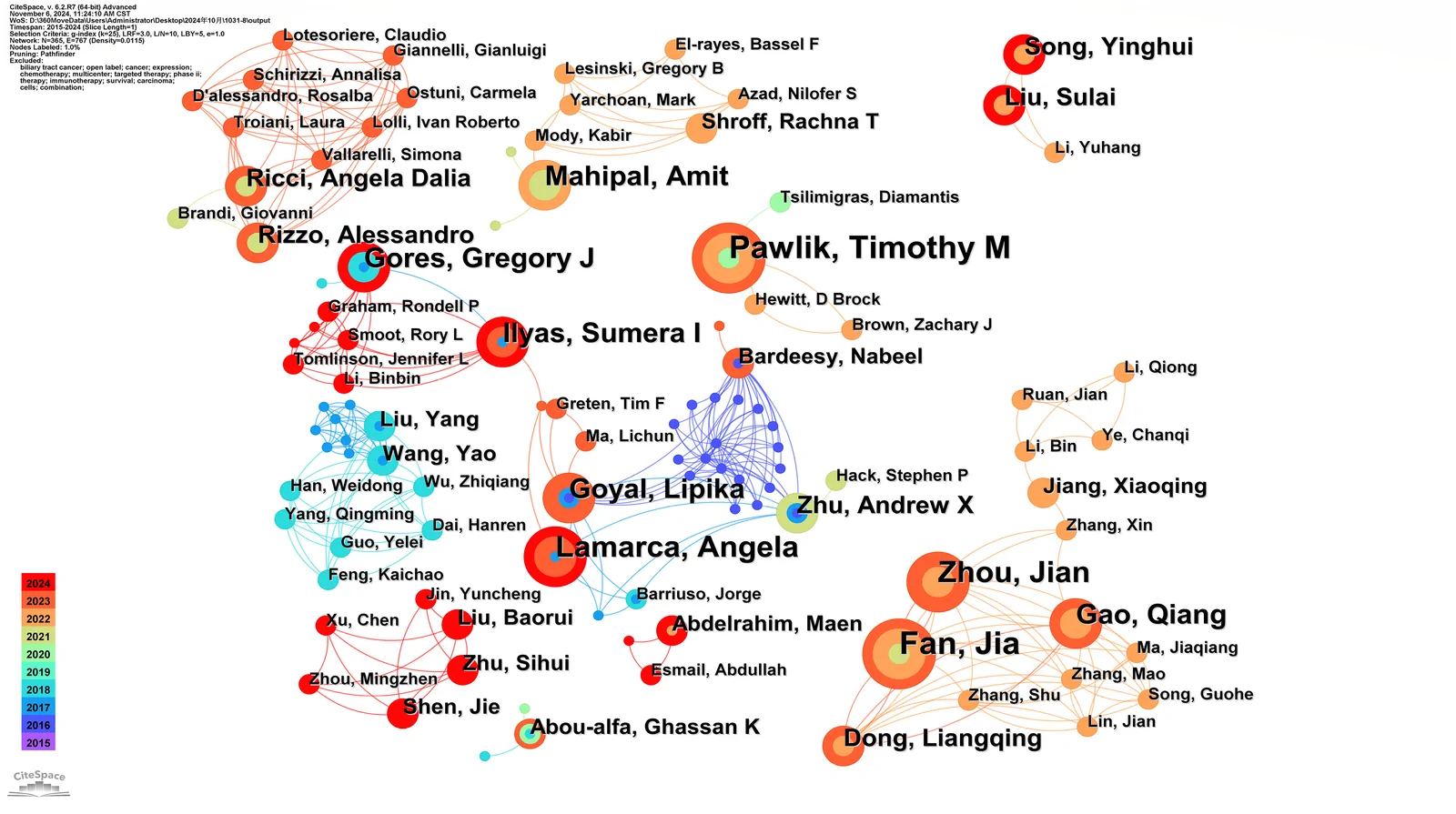
Author map researching immunotherapy for biliary tract cancers from 2014 to 2024.

### 3.4. Analysis of co-cited references

By employing a counts and centrality study, we presented the top 5 co-cited references (Fig. 6, Table 3). As can be seen from Table 3, two of the articles were published by one author. Among them, the paper entitled “Efficacy and safety of pembrolizumab for the treatment of advanced biliary cancer: Results from the KEYNOTE-158 and KEYNOTE-028 studies” published by Piha-Paul SA (2020) et al had the most co-citations (n = 106).

**Table 3:** Top 5 co-cited references related to immunotherapy for biliary tract cancers in terms of co-citation.

Ranking	Cited reference	Journal	Co-citation counts	Representative author (publication year)	DOI
1	Efficacy and safety of pembrolizumab for the treatment of advanced biliary cancer: Results from the KEYNOTE-158 and KEYNOTE-028 studies	Int J Cancer	106	Piha-Paul SA (2020)	10.1002/ijc.33013
2	A phase 2 multi-institutional study of Nivolumab for patients with advanced refractory biliary tract cancer	JAMA Oncol	104	Kim RD (2020)	10.1001/jamaoncol.2020.0930
3	Cholangiocarcinoma 2020: the next horizon in mechanisms and management	Nat Rev Gastro Hepat	104	Banales JM (2020)	10.1038/s41575-020-0310-z
4	Pemigatinib for previously treated, locally advanced or metastatic cholangiocarcinoma: a multicentre, open-label, phase 2 study	Lancet Oncol	103	Abou-Alfa GK (2020)	10.1016/S1470-2045(20)30109-1
5	Ivosidenib in IDH1-mutant, chemotherapy-refractory cholangiocarcinoma (ClarIDHy): a multicentre, randomized, double-blind, placebo-controlled, phase 3 study	Lancet Oncol	87	Abou-Alfa GK (2020)	10.1016/S1470-2045(20)30157-1

DOI = digital object identifier.

**Figure 6.**
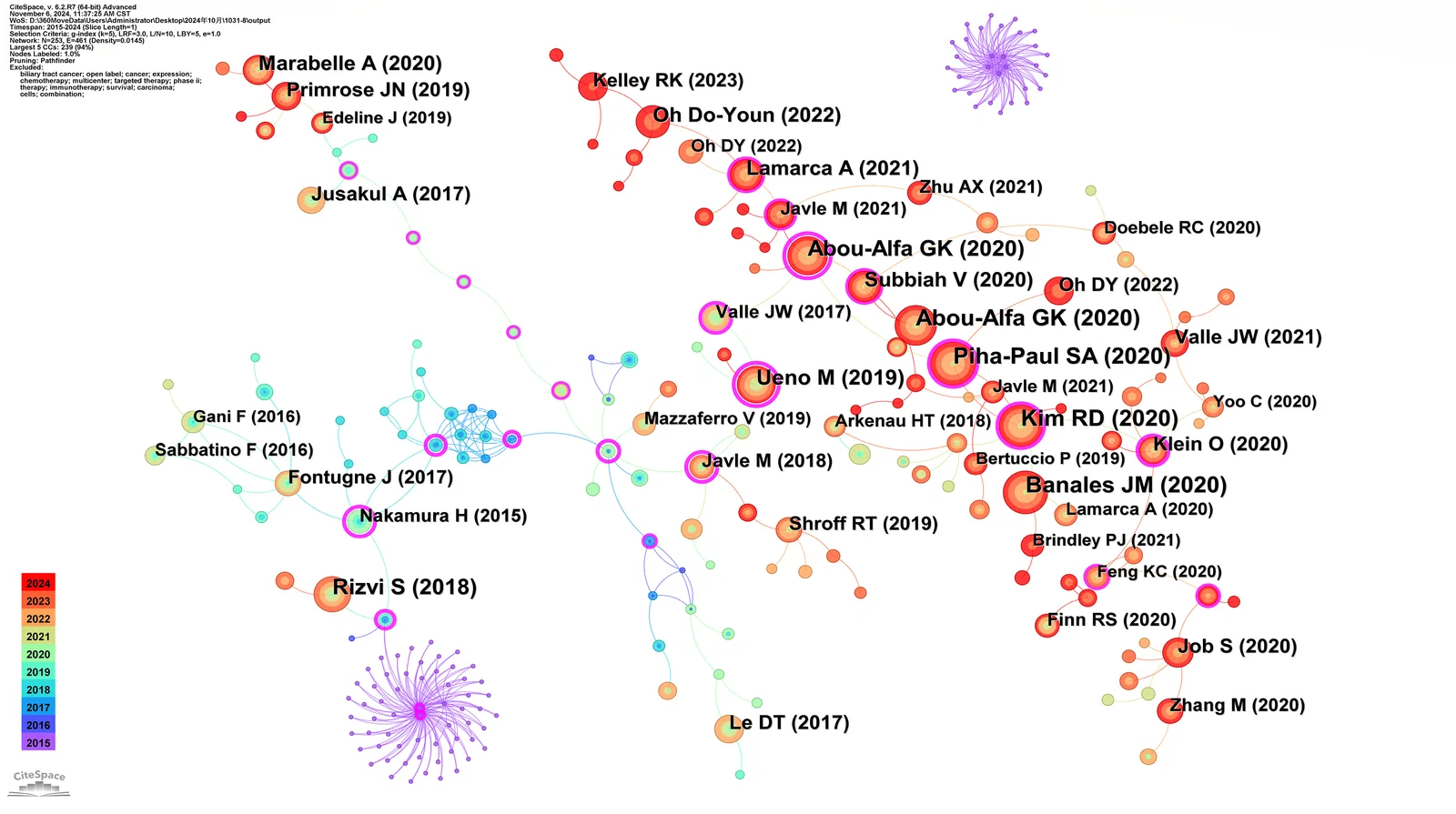
Co-cited references map researching immunotherapy for biliary tract cancers from 2014 to 2024.

### 3.5. Analysis of keyword co-occurrence and burst keywords analysis

The created phrase co-occurrence map has 318 nodes and 643 linkages; a keyword co-occurrence map might show trending subjects (Fig. 7). “intrahepatic cholangiocarcinoma, gemcitabine, hepatocellular carcinoma, tumor microenvironment, t cells” were the top 5 keywords. These can also correspond to highly cited literature. There’s a lot of interest in gemcitabine and tumor microenvironment. The standard of therapy for patients with advanced BTC who do not exhibit targetable alterations is still gemcitabine plus cisplatin in the first-line scenario. Furthermore, for first-line chemotherapy, gemcitabine + S − 1 (G S − 1) or capecitabine + oxaliplatin (XELOX) are advised,^[1,17]^ despite these significant developments, the tumor microenvironment still requires improved molecular and cellular characterization since it plays a critical role in the onset and spread of biliary tract cancer.^[18]^

**Figure 7.**
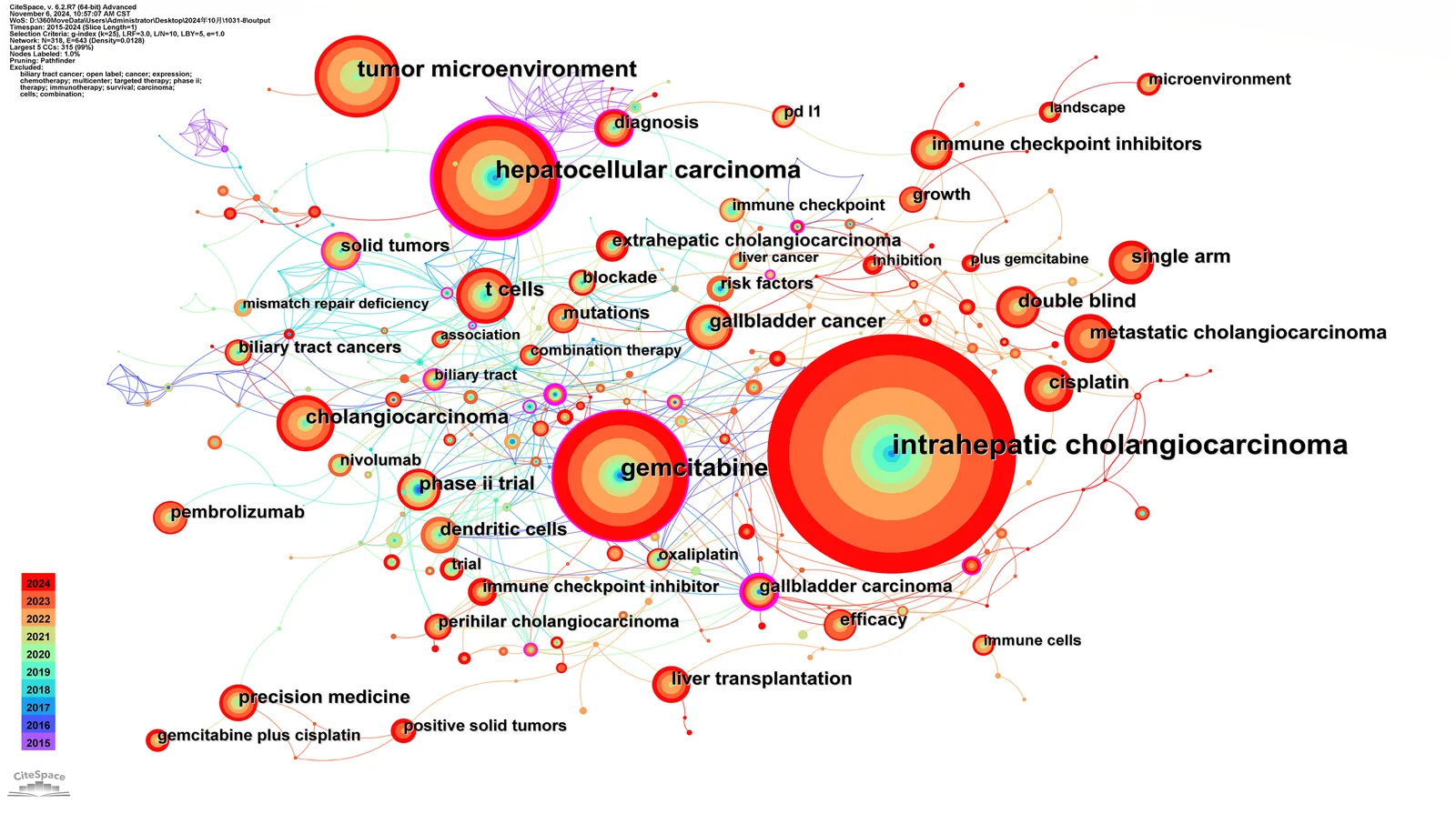
Co-occurrence keyword map researching immunotherapy for biliary tract cancers from 2014 to 2024.

“Burst words” are those that are used a lot and over long stretches of time. By examining the distribution of terms with the largest citation explosion, it may be feasible to forecast the course of future study. Figure 8 displays the top 10 keywords with the highest citation increase from 2014 to 2024. The green bars show how frequently the keyword was referenced, while the red bars show how frequently it was cited. The frequent usage of terms like “tumor infiltrating lymphocytes,” “lymphocytes,” “cell lung cancer,” “growth factor receptor,” and “adoptive immunotherapy” suggests that future research in those areas will probably be given more attention.

**Figure 8.**
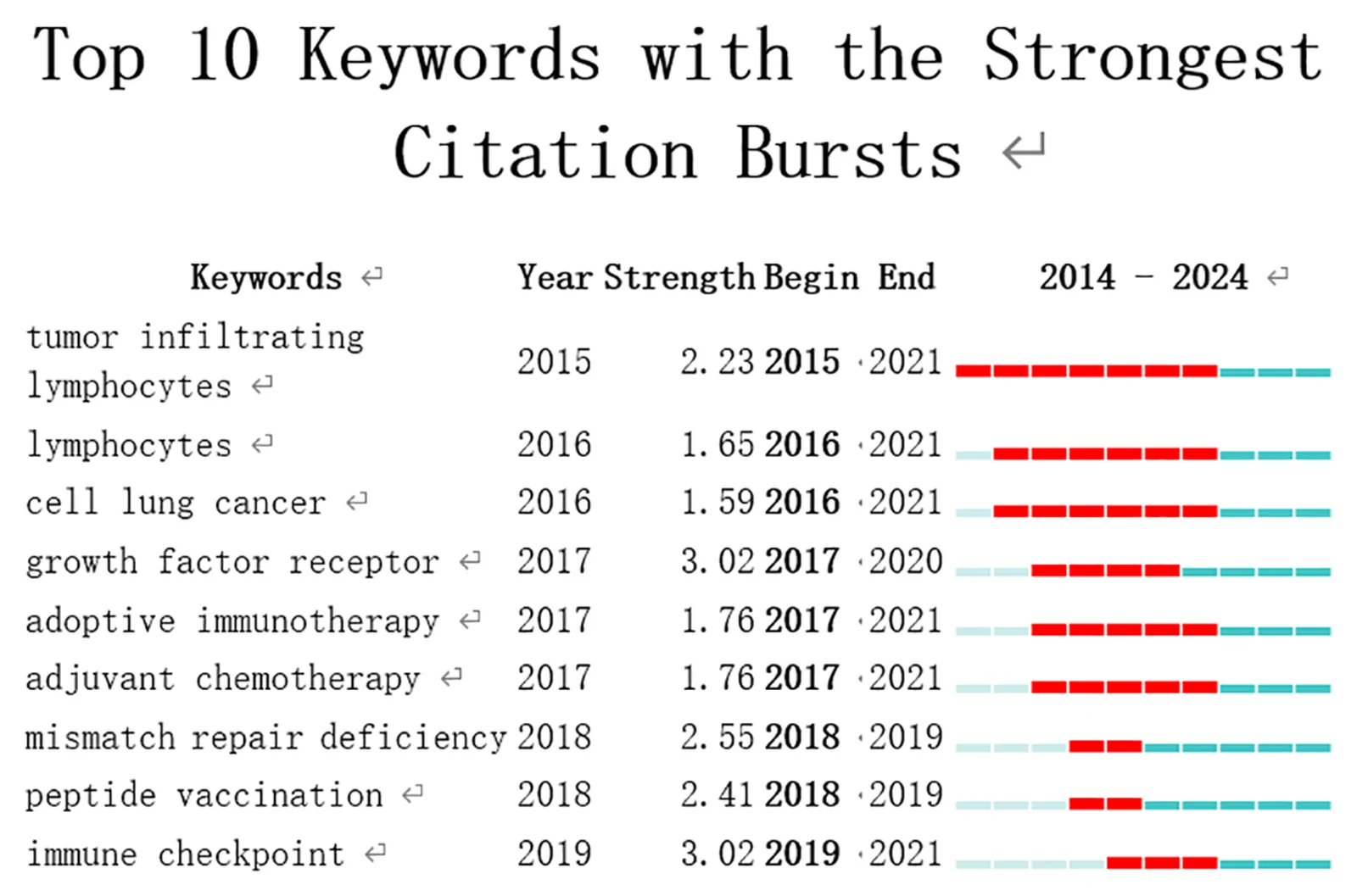
Top 10 keywords with the strongest citation bursts.

## 4. Discussion

CiteSpace data shows that from 2021 to 2024, the annual publication rate of papers about immunotherapy for BTC grew. This trend may be explained by researchers focusing more on the use of immunotherapy in BTC cases, which may be connected to important developments in the field of BTC immunotherapy. We found that the top 5 cited articles were all published in 2020.^[19-23]^. These articles have a strong influence and may have contributed to the rapid increase in the number of BTC publications in the field of immunotherapy in 2021.

Even though the reported prevalence of BTC is higher among American Indians and Latin American people, China is the nation with the most publications on immunotherapy for BTC, which is noteworthy because it is the only developing nation on this list.^[24]^ The epidemiology of BTC in 2009 identified Shanghai, China (5.2/1,00,000 women) as a high-risk area.^[4]^ Fudan University in Shanghai, China, as the number 1 institution in terms of publications, also proves this point. Fan, Jia, as the most published person, is employed at Zhongshan Hospital Affiliated with Fudan University in Shanghai.

From the information on keyword co-occurrence, we found that “gemcitabine, tumor microenvironment, t cell” are highly ranked. Although no has ever been sufficiently standard palliative regimen, gemcitabine^[25,26]^ have shown activity. Oncologists who specialize in hepatobiliary illness have increasingly prescribed gemcitabine as a therapy for biliary tract malignancy. When used with gemcitabine, cisplatin has been shown to have an additive or synergistic impact on a variety of tumor forms.^[27-29]^, it was the initial treatment for cancer of the biliary system. The noncancerous cells and other elements found in the tumor, as well as the chemicals they create and release, are referred to as the “tumor microenvironment” (TME). The genesis, development, metastasis, and response to treatment of tumors are significantly influenced by the ongoing interactions between cancer cells and the tumor microenvironment. In both research and treatment contexts, the tumor microenvironment has grown in popularity as a target for cancer treatment.^[30]^ Targeting TME components to provide therapeutic advantages for cancer patients has been the subject of various studies as awareness of the critical functions the TME plays in tumor progression and treatment resistance has increased.^[30]^ Adaptive immunity relies heavily on effector T cells. Immunologists have made significant progress in recent years in their comprehension of the properties of T cells in patients’ tumor settings,^[31-33]^ however, a thorough knowledge of tumor-resident T cells is necessary to develop innovative cancer immunotherapies and identify useful biomarkers.^[34]^

Burst keyword analysis revealed tumor-infiltrating lymphocytes, growth factor receptor, and adoptive immunotherapy were the burst keywords for the past 10 years. According to some research, tumor-infiltrating lymphocytes, which are biological mediators of the adaptive immune response like CD8+ and CD4+ cells, are often associated with improved outcomes in BTC. It was the justification for immunotherapy’s potential efficacy in Bitcoin.^[35]^ The paradigm of therapy has changed in recent years due to targeted medicines (growth factor receptor-2).^[1]^ In certain individuals with biliary tract cancer, overexpression, amplification, or mutation can activate the epidermal growth factor receptor family member receptor tyrosine-protein kinase erbB-2 (ERBB2; HER2), according to some research, which has confirmed that preclinical models of BTC have an upregulation of the epidermal growth factor receptor (EGFR) pathway. Up to 15% to 20% of instances of gallbladder cancer and extrahepatic cholangiocarcinoma may have ERBB2 overexpression or gene amplification, but intrahepatic cholangiocarcinoma has significantly lower activation rates.^[28,36]^ Further study is needed to determine if ERBB2-targeted therapies, either alone or in combination, are beneficial for patients with ERBB2-activated biliary tract cancer.

## 5. Limitations

Analysis and visualization tools such as CiteSpace offer a comprehensive picture of the evolution and trajectory of immunotherapy for BTC. Nonetheless, this study has several inherent limitations. The scope of the literature considered is limited, as it only includes data from Web of Science and excludes information from other relevant databases. Because only English-language publications are included in the literature selection, there is also linguistic bias. Therefore, it is probable that the literature analysis may not fully cover all studies pertinent to immunotherapy.

## 6. Conclusion

With excellent publishing rates and centrality, the People’s Republic of China, Fudan University, and Fan, Jia have emerged as the 3 key research nations/institution/author in this discipline. Research on immunotherapy for BTC advances when developed nations and prestigious organizations form the strongest alliances. These articles were often cited because they provide guidance or have a high impact factor. Some areas of intense investigation in this discipline are gemcitabine, growth factor receptor, tumor microenvironment, and t cells.

## Acknowledgment

The authors would like to thank Professor Chaomei Chen for creating CiteSpace and allowing anybody to use it for free.

## Author contributions

**Conceptualization:** Yanyan Miu, Zhongbiao Nie.

**Data curation:** Yanyan Miu.

**Funding acquisition:** Zhongbiao Nie.

**Investigation:** Cuiling Zhang.

**Methodology:** Yanyan Miu, Cuiling Zhang, Peirong Wang.

**Project administration:** Yan Niu, Peirong Wang.

**Resources:** Yan Niu, Zhongbiao Nie.

**Writing – review & editing:** Zhongbiao Nie.
